# Poststroke Depression Biomarkers: A Narrative Review

**DOI:** 10.3389/fneur.2018.00577

**Published:** 2018-07-16

**Authors:** Oleg A. Levada, Alexandra S. Troyan

**Affiliations:** State Institution “Zaporizhzhia Medical Academy of Postgraduate Education Ministry of Health of Ukraine”, Zaporizhzhia, Ukraine

**Keywords:** poststroke depression, neuroimaging biomarkers, molecular biomarkers, neurophysiological biomarkers, diagnosis

## Abstract

Poststroke depression (PSD) is the most prevalent psychiatric disorder after stroke, which is independently correlated with negative clinical outcome. The identification of specific biomarkers could help to increase the sensitivity of PSD diagnosis and elucidate its pathophysiological mechanisms. The aim of current study was to review and summarize literature exploring potential biomarkers for PSD diagnosis. The PubMed database was searched for papers published in English from October 1977 to December 2017, 90 of which met inclusion criteria for clinical studies related to PSD biomarkers. PSD biomarkers were subdivided into neuroimaging, molecular, and neurophysiological. Some of them could be recommended to support PSD diagnosing. According to the data, lesions affecting the frontal-subcortical circles of mood regulation (prefrontal cortex, basal nuclei, and thalamus) predominantly in the left hemisphere can be considered as neuroimaging markers and predictors for PSD for at least 1 year after stroke. Additional pontine and lobar cerebral microbleeds in acute stroke patients, as well as severe microvascular lesions of the brain, increase the likelihood of PSD. The following molecular candidates can help to differentiate PSD patients from non-depressed stroke subjects: decreased serum BDNF concentrations; increased early markers of inflammation (high-sensitivity C-reactive protein, ferritin, neopterin, and glutamate), serum pro-inflammatory cytokines (TNF-α, IL-1β, IL-6, IL-18, IFN-γ), as well as pro-inflammatory/anti-inflammatory ratios (TNF-α/IL-10, IL-1β/IL-10, IL-6/IL-10, IL-18/IL-10, IFN-γ/IL-10); lowered complement expression; decreased serum vitamin D levels; hypercortisolemia and blunted cortisol awakening response; S/S 5-HTTLPR, STin2 9/12, and 12/12 genotypes of the serotonin transporter gene SLC6A4, 5-HTR2a 1438 A/A, and BDNF met/met genotypes; higher SLC6A4 promoter and BDNF promoter methylation status. Neurophysiological markers of PSD, that reflect a violation of perception and cognitive processing, are the elongation of the latency of N200, P300, and N400, as well as the decrease in the P300 and N400 amplitude of the event-related potentials. The selected panel of biomarkers may be useful for paraclinical underpinning of PSD diagnosis, clarifying various aspects of its multifactorial pathogenesis, optimizing therapeutic interventions, and assessing treatment effectiveness.

## Introduction

Poststroke depression (PSD) is the most prevalent psychiatric disorder after stroke, which affects nearly one-third of the survivors during first 5 years after disease onset (1–3). The diagnosis of PSD includes the following characteristics: (1) presence of major/minor depressive episode according to DSM-III-IV-5 or other valid approaches; (2) evidence of stroke from history, physical examination, and/or neuroimaging data; and (3) onset of PSD is temporally related to the stroke (3). Several epidemiological findings have demonstrated that PSD is independently linked to negative clinical outcomes, such as significantly longer hospitalization; more severe functional disability (3–6); profound diminutions in physical, psycho-social, cognitive, and eco-social domains of quality of life (3, 7); unsatisfactory results of poststroke rehabilitation (8); elevated rates of mortality (3, 9–11); higher risks of recurrent stroke at 1 year (12); as well as considerable strain for caregivers (13). Data mentioned above highlight the importance of identifying PSD among stroke survivors.

The detection of depressive symptoms at early stroke stages and recognition subjects at risk for PSD diagnosis remains challenging. Clinical measures currently used to assess PSD, especially in the acute poststroke patients, may lack the specificity necessary to detect symptoms (14, 15). From this point of view, the identification of specific biomarkers might help to increase the sensitivity of PSD diagnosis. Moreover, it could be helpful for elucidating the pathophysiological mechanisms of PSD and ultimately lead to choosing specific targeted treatment (16).

Thus, we aimed to review and summarize the literature exploring potential biomarkers for PSD diagnosis.

## Methods

We searched PubMed database for studies published in English using keywords: “poststroke depression” and “depression after stroke.” The search covered a period from October 1977 to December 2017. We also reviewed the reference lists of obtained articles for additional information. Further, human clinical articles related to biomarkers of PSD were subjected to a comprehensive analysis. The inclusion criteria were: (1) peer-reviewed original studies with case-control design and all types of reviews, where the relationship between PSD and possible biomarkers was studied; (2) age of participants ≥ 18 years; (3) patients had ischemic or hemorrhage stroke at the time of entry; (4) valid instruments for PSD assessment; (5) standardized measurements for biomarkers. We excluded duplicate articles with the same data set and studies without sufficient data. All articles were reviewed and analyzed by the first author (O.L.). Received results were checked for accuracy by the second investigator (A.T.). Discrepancies if occurred were resolved by discussion and consensus.

## Results and discussion

**Figure 1:**
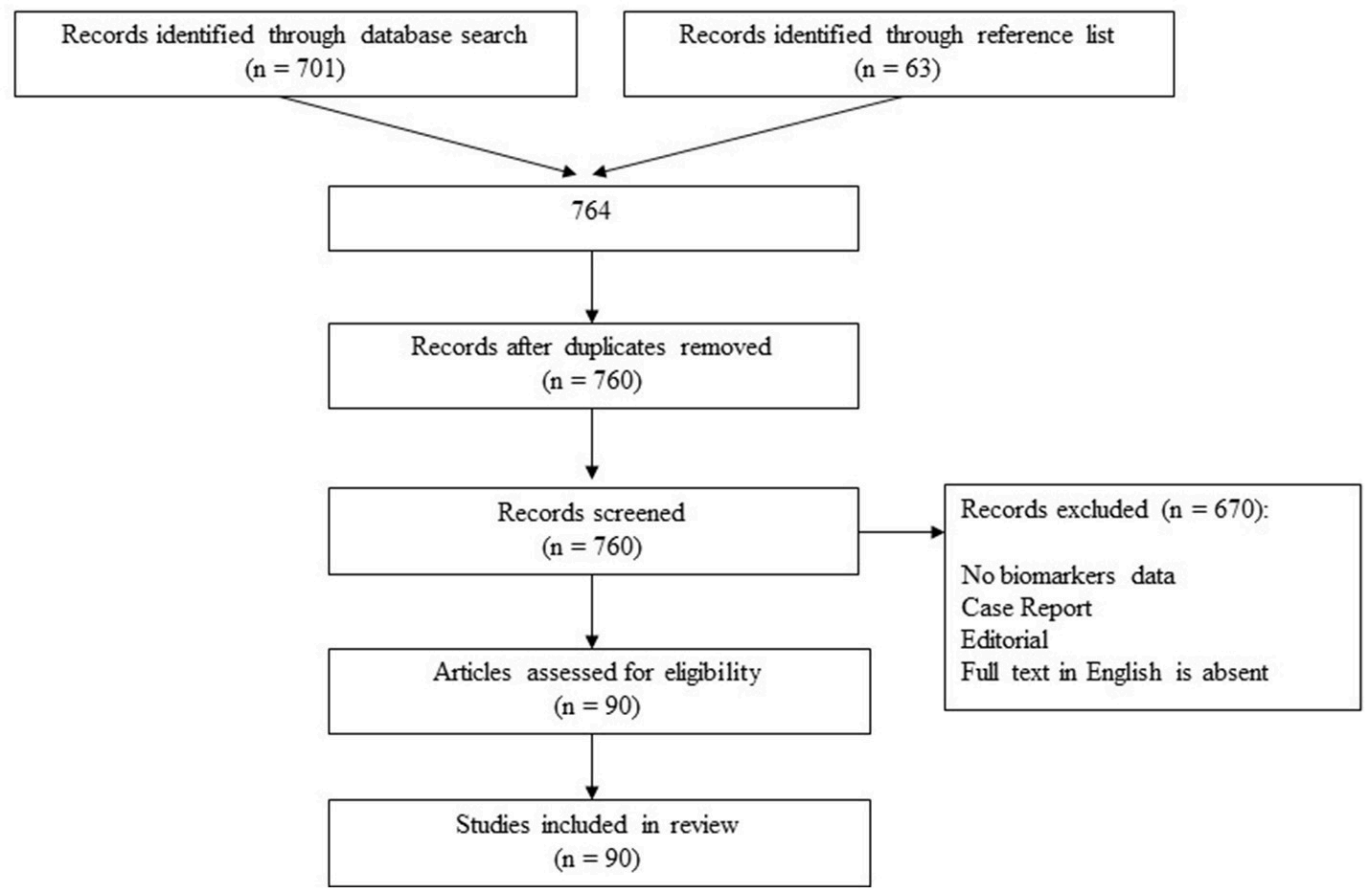
Study selection process.

At the first stage, 764 clinical and experimental articles were identified, 90 of which met inclusion criteria and underwent a detailed analysis at the second stage (Figure 1). The results of included studies were subdivided according to the type of investigated biomarkers into neuroimaging, molecular, and neurophysiological. In turn, molecular PSD biomarkers were categorized into monoamines, growth factors, markers of neuroinflammation, markers of the hypothalamic-pituitary-adrenal axis, markers of oxidative damage, other metabolites, and genetic markers for convenient structural representation of the data obtained.

## Neuroimaging biomarkers of PSD

In spite of being one of the straight roots of depression, a question if a stroke-determined neuroanatomical substrate is actually contributing to the development of PSD remains a matter of debate (17, 18). The results of neuroimaging research in PSD patients are presented in Table 1. Methodological peculiarities of the main studies allow having an idea of the importance of clinical-anatomical relationships in this cohort of patients.

**Table 1:** Summary of neuroimaging biomarkers of PSD.

**Study**	**Type of study**	**Patients**	***N***	**Depression instrument**	**Results**
House et al. (19)	Prospective	Poststroke	73	n/a	A weak correlation between PSD severity and the proximity of the stroke lesion to the frontal pole of the hemisphere, but no evidence of a difference between right and left hemisphere strokes in the nature of the relationship between lesion distribution and mood symptoms
Paolucci et al. (20)	Prospective	PSD Non-PSD	129 341	n/a	No association between the lesion site and PSD
Berg et al. (21)	Prospective	PSD Non-PSD	54 46	DSM-III-R, BDI, HDRS	No differences in PSD prevalence between right- and left-hemispheric lesions
Gozzi et al. (22)	Prospective	PSD Non-PSD	15 55	HADS, MINI, DSM-IV	Minimal overlap of infarct location between patients. No voxels/clusters were significant after a multiple comparison correction (*P* > 0.05)
Murakami et al. (23)	Prospective	Poststroke	149	HADS	In patients with higher depressive scores, the lesion overlap centered on the brainstem, left basal ganglia, and left frontal cortex
Narushima et al. (24)	Meta-analysis	With left hemisphere stroke With right hemisphere stroke	163 106	n/a	The inverse correlation between the severity of depressive symptoms and the distance of the anterior border of the lesion from the frontal pole (Z = −7.04, *P* < 0.001 on the fixed model, Z = −4.68, *P* < 0.001 on the random model). The correlation in the left hemisphere was higher than that of the right hemisphere (Z fixed = 3.43, *P* < 0.0001, Z random = 2.63, *P* < 0.008)
Parikh et al. (25)	Prospective	Poststroke	103	HDRS	A strong positive correlation between proximity of the lesion to the frontal pole in patients with left anterior infarcts with PSD severity at 1 year (*r* = 0.76)
Starkstein et al. (26)	Prospective	Poststroke	25	HDRS-17	A significantly higher frequency and severity of PSD in patients with left-sided basal ganglia lesions, as compared with patients with right-sided basal ganglia or thalamic (left or right-sided) lesions
Morris et al. (27)	Prospective	Poststroke	41	MADRS	Patients with lesions in left hemisphere prefrontal or basal ganglia had a higher frequency of PSD (75%) than other left (8%) or right hemisphere lesions (29%), *P* = 0.002
Zhang et al. (28)	Case-control	PSD Non-PSD HC	26 24 24	HDRS	In PSD patients functional connectivity of the affective network was disrupted compared to non-PSD and HC. The left orbital part of inferior frontal gyrus significantly correlated with HDRS scores in PSD patients (*r* = 0.39, *P* = 0.05)
Yang et al. (17)	Prospective	PSD Non-PSD	40 76	HAMD-24	17 nodes were selected to build the depression-related subnetwork. Decreased local efficiency of the subnetwork was a significant risk factor for PSD (RR:0.84, 95% CI:0.72–0.98, *P* = 0.027)
Nys et al. (29)	Cross-sectional	PSD Non-PSD	66 60	MADRS	*Post-hoc* Scheffe' analysis revealed that PSD patients with moderate to severe depressive symptoms had a larger lesion volume than non-PSD (*P* = 0.008) or PSD with mild depressive symptoms (*P* = 0.05). No association was found between PSD severity and lesion location
Bhogal et al. (30)	Review	26 reports	n/a	HDRS, ZSDS	Within the first 28 days of stroke, a significant association between left hemisphere stroke and PSD was demonstrated (OR:2.14, 95% CI:1.50–3.04). Studies that assessed depression between 1 and 4 months showed that the association began to favor development of PSD after right hemisphere stroke (OR:0.93, 95% CI:0.66–1.32), with a significant association between right hemisphere stroke and PSD at 6 months (OR:0.53, 95% CI:0.30–0.93)
Levada et al. (31)	Review	n/a	n/a	n/a	In acute stage and 3–6 months after stroke, PSD is associated with left-hemisphere lesion severity and proximity of the lesion to the frontal pole and related to the dysfunction of (cortico-) striato-pallido-thalamic-cortical projections. In post-stroke period (1–2 years) PSD is associated with right-hemisphere lesion severity and proximity of the lesion to the occipital pole
Wongwandee et al. (32)	Cross-sectional	PSD Non-PSD	11 28	HDRS	Left sided stroke lesion is one of the factors significantly related to early onset of PSD
21 Omura et al. (8)	Prospective	PSD Non-PSD	41 134	PHQ-9	Lesions in the right and left thalami were significantly and independently associated with PSD in acute phase of stroke: left thalamus OR = 5.99 (95% CI:1.44–25.01, *P* = 0.01); right thalamus OR = 5.32 (95% CI: 1.29–21.99, *P* = 0.02)
Nishiyama et al. (33)	Prospective	PSD Non-PSD	46 88	ZSDS	Left lenticulocapsular lesions were significantly associated with PSD occurrence at 1 month after stroke (OR:4.30, 95% CI:1.10–16.90)
Herrmann et al. (34)	Cross-sectional	PSD Non-PSD	30 17	MADRS, DSM-III-R	Within a 2-month period after the acute stroke, patients with lesions of the left hemisphere basal ganglia or lesions in the left hemisphere lenticulostriate or anterior choroidal artery area of vascular supply showed a higher frequency of PSD (χ^2^ = 10.7, df = 1, *P* < 0.01) and scored higher on MADRS (CDS, Mann-Whitney U = 114.5, *P* < 0.001; MAS, Mann-Whitney U = 60, *P* < 0.01) compared with patients with other lesions
Lassalle-Lagadec et al. (35)	Prospective	Poststroke	32	HDRS	PSD severity at 3-month follow-up correlated positively with frontal connectivity index in left middle temporal cortex (*R* = 0.77, 95% CI:0.51–0.90, *P* = 0.001) and left precuneus (*R* = 0.76, 95% CI: 0.49–0.89, *P* = 0.001) and negatively with left neostriatum (*R* = 20.77, 95% CI: 20.90–20.50, *P* = 0.001)
Tang et al. (36)	Prospective	PSD Non-PSD	75 516	DSM-IV	Frontal subcortical circuits infarcts predict PSD with OR 2.57 (95% CI:1.30–5.07, *P* = 0.007)
Vataja et al. (37)	Prospective	PSD Non-PSD	109 166	DSM-III-R, DSM-IV	Patients with depression had a higher number and larger volume of infarcts affecting the prefrontosubcortical circuits, especially the caudate, pallidum, and genu of internal capsule, with left-sided predominance. Independent correlates of PSD in a logistic regression model were mean frequency of infarcts in the genu of internal capsule on the left side (OR:3.2; 95% CI:1.0–10.1)
Tang et al. (38)	Prospective	PSD Non-PSD	27 158	Structured Clinical Interview for DSM-IV	Multivariate logistic regression analysis showed subcortical infarcts and anterior cerebral artery lesions were independent risk factors for PSD with OR = 1.81 (95% CI:1.08–3.04, *P* = 0.025) and OR = 6.46 (95% CI:1.62–25.78, *P* = 0.008), respectively
Vataja et al. (39)	Cross-sectional	PSD Non-PSD	62 96	MADRS	In the logistic models, the only correlate for depression–dysexecutive syndrome was number of brain infarcts affecting frontal-subcortical circuits on the left side (OR:1.6, 95% CI:1.1–2.5, *P* = 0.02)
Beblo et al. (40)	Cross-sectional	PSD	20	DSM-III-R	For both major and minor depression the maximal overlap of lesions was found in subcortical areas, including parts of the caudate nucleus, posterior parts of the putamen, and the deep white matter
Kim et al. (41)	Prospective	PSD Non-PSD	27 121	DSM-IV, BDI	The presence of PSD was more frequently associated with anterior cortical than posterior cortical lesions (*P* < 0.01)
Singh et al. (42)	Longitudinal	PSD Non-PSD	52 29	MADRS, ZSDS	A highly significant model was produced, with 1-month covariate data predicting depression scores at 3 months [*F*_(3, 76)_ = 9.8, *P* < 0.0005], accounting for 28% of the variance. The significant variable contributing to this model was damage to the inferior frontal region. Regression performed with only the 15 CT regions revealed that damage to the inferior frontal region still emerged as the only significant CT variable (*P* < 0.001, standardized β = 0.36) predicting depression scores, accounting for 13% of the model variance
Vataja et al. (43)	Prospective	PSD Non-PSD	26 44	DSM-III-R, DSM-IV	The only independent correlate of PSD was the infarct location in the pallidum (OR:7.2, 95% CI:1.9–35.7, SE = 0.73, coefficient:1.9)
Lauterbach et al. (44)	Case-control	With focal subcortical lesions	45	DSM-III, DSM -III-R, DSM -IV	Pallidal lesions were present in eight (89%) of 9 subjects with PSD and 13 (59%) of 22 controls. Left posterior pallidal lesions occurred in four (44%) of the nine subjects with PSD and two (9%) of the 22 controls (one-tailed Fisher's exact test *P* = 0.043). Abnormal pallidal function may contribute to depressive pathophysiology, perhaps by influencing basal ganglia-thalamocortical mood circuits. Left-lateralized circuits in the posterior pallidum may be of particular relevance
Zhang et al. (18)	Meta-analysis of 31 reports	Poststroke	5,309	n/a	The pooled OR of PSD after a left-hemisphere stroke, compared with a right-hemisphere stroke was 1.11 (95% CI:0.82–1.49). Subacute poststroke subgroup (1–6 months) significantly favored PSD occurring after a left hemisphere stroke (OR:1.50, 95% CI:1.21–1.87)
Grajny et al. (45)	Cross-sectional	With left hemisphere stroke	39	SADQ	Lesions in the dorsolateral prefrontal cortex (DLPFC) were associated with increased severity of PSD in chronic left hemisphere stroke survivors. The analysis identified a single significant cluster centered in the left DLPFC in which lesions were associated with greater depression symptoms (volume = 1.24 mm^3^; peak MNI coordinates −33, 21, 26; center −42, 26, 20). Independent ANOVAs demonstrated that, controlling for education and total lesion volume, SADQ scores related to DLPFC damage (>1/2 of the voxels in the SVR-LSM cluster lesioned) in both groups (moderate-severe comprehension deficit group, *F*_(1, 8)_ = 17.09, *P* = 0.003; mild-to-no comprehension deficit group, *F*_(1, 23)_ = 9.49, *P* = 0.005)
Tang et al. (46)	Prospective	PSD Non-PSD	75 154	GDS-15	Compared to non-PSD, patients with PSD were more likely to have pontine cerebral microbleeds (32.0% vs. 18.2%, *P* = 0.019). The presence of pontine cerebral microbleeds remained an independent predictor of PSD in the multivariate analysis (OR:2.2, 95% CI:1.2–4.2, *P* = 0.016)
Tang et al. (47)	Prospective	Nonremitters PSD Remitters PSD	89 46	GDS-15	At 1-year follow-up, PSD nonremitters compared to PSD remitters were more likely to have lobar cerebral microbleeds (18.4% vs. 4.3%, *P* = 0.02). Lobar cerebral microbleeds remained an independent predictor of PSD in the multivariate analysis (OR:4.96, *P* = 0.04)
Tang et al (48)	Prospective	PSD Non-PSD	84 151	GDS-15	In comparison with non-PSD, PSD patients were more likely to have lobar cerebral microbleeds (33.3% vs. 19.9%; *P* = 0.02). Lobar cerebral microbleeds remained an independent predictor of PSD at 3 months after stroke in the multivariate analysis (OR:2.09, 95% CI:1.07–4.11, *P* = 0.03)
Provinciali et al. (49)	Observational	PSD Non-PSD	264 467	BDI, MADRS	Only patients with total anterior cerebral ischemia showed a higher PSD occurrence (OR:1.76, CI:1.14–2.71)
Arba et al. (50)	Retrospective	Lacunar infarcts Other	1127 1033	HADS	In the adjusted multivariable models lacunar stroke subtype was independently associated with reduced risk of depression (OR:0.71, 95% CI:0.55–0.93) compared to other stroke types
Santos et al. (51)	Neuropathological	PSD Non-PSD	20 21	DSM-IV	Basal ganglia and thalamic lacunes were the best neuropathological correlates of PSD; the higher the lacunar scores in these areas, the greater the risk of PSD (Mann-Whitney U test, z = −3.129; *P* = 0.002). Semiquantitative severity scores for white matter lacunes were also related to the occurrence of PSD (Mann-Whitney *U*-test, z = −2.211; *P* = 0.03). This was not the case for microinfarcts nor for diffuse or periventricular white matter demyelination (Mann-Whitney *U*-test, z = −0.425, probability values of 0.67, z = −0.705, 0.48, and z = 0.087, 0.93, respectively). The combined brain lacune score (thalamic plus basal ganglia plus deep white matter) was strongly related to the occurrence of PSD (*P* = 0.001) and explained 25% of the variability of this occurrence. In regression models, basal ganglia and thalamic lacunes explained 20% of the variability in PSD occurrence, whereas deep white matter lacunes added an extra 5%
Chen et al. (52)	Prospective	PSD Non-PSD	44 83	GDS-15	PSD group had a higher percentage of large artery disease (LAD) cases (52.3% vs. 25.3%, *P* = 0.002). In the regression model, the etiological type LAD was a significant independent risk factor (OR:3.25, *P* = 0.003) for PSD at 3 months after stroke. Multiple acute infarcts (*P* < 0.001) were significantly more frequent in the LAD group. There was a higher number of acute infarcts and a higher number of total infarcts in LAD group (*P* < 0.05). The LAD group also had a higher frequency of superficial infarcts (27.3 vs. 12.0%, *P =* 0.03)
Liang et al. (53)	Prospective	PSD Non-PSD	153 572	GDS-15	Centrum semiovale enlarged perivascular space (CS-EPVS) continuous score was an independent predictor of PSD at 3 months stroke (OR:1.27, 95% CI:1.03–1.57). After dichotomized, moderate to severe CS-EPVS was independently associated with PSD (OR:1.68, 95% CI:1.10–2.57)
Kim et al. (54)	Prospective	PSD Non-PSD	63 70	HADS	The independent predictor of depressive symptoms at 3 months were deep white matter hyperintensities (OR:4.051, 95% CI:1.19–13.75, *P* = 0.025)
Chen et al. (55)	Prospective	Acute: PSD Non-PSD 3 mths: PSD Non-PSD	85 122 73 120	HDRS-24	In acute phase: PSD patients had more frequent intracranial atherosclerosis (56.5 vs. 38.5%, *P* = 0.01). Multiple logistic regression analysis revealed that intracranial atherosclerosis (OR:1.9, 95% CI:1.03–3.5, *P* < 0.001) was independently and significantly associated with PSD. At 3 months: PSD patients had a higher frequency of intracranial atherosclerosis (61.6 vs. 38.3%, *P* = 0.002). Logistic regression analysis showed intracranial atherosclerosis (OR:2.1, 95% CI:1.09–4.0, *P* = 0.02) was independently and significantly associated with PSD
Noonan et al. (56)	Meta-analysis	Poststroke	1893	DSM, 9 mood scales	Moderate effect was detected for smaller amygdala volumes (SMD −0.45, 95% CI: −0.89 to −0.02, *P* = 0.04), and a small effect for overall brain perfusion reduction (SMD −0.35, 95% CI: −0.64 to −0.06, *P* = 0.02) on poststroke depression
Stern-Nezer et al. (57)	Prospective	PSD Non-PSD	13 76	HDRS	Depression was not associated with hematoma volumes and presence of intraventricular hemorrhage: mean hematoma volume (cc3) (median, IQR) for PSD 14 (8.5–39.5), whereas for non-PSD 10 (3–35.5), *P* = 0.45; presence of intraventricular hemorrhage in 3 PSD patients (23.1%) and in 29 non-PSD patients (38.2%), *P* = 0.3

*BDI, Beck Depression Inventory; CI, confidence interval; DSM, Diagnostic and Statistical Manual; GDS-15, 15-item Geriatric Depression Scale; HADS, Hospital Anxiety and Depression Scale; HC, healthy controls; HDRS, Hamilton Depression Rating Scale; IQR, interquartile range; MADRS, Montgomery and Asberg Depression Rating Scale; MINI, Mini-International Neuropsychiatric Interview; Non-PSD, poststroke patients without depression; OR, odds ratio; PSD, poststroke patients with depression; RR, relative risk; SADQ, Stroke Depression Questionnaire; SMD, standardized mean difference; ZSDS, Zung Self Report Depression Questionnaire*.

The results of some well-organized studies using different MRI/CT techniques do not yield evidence that lesions in a distinct neuroanatomical region induce PSD (19–22). In contrast, a large number of clinical-neuroimaging investigations found significant associations between stroke location and PSD. Murakami et al. revealed that PSD was associated with infarcts located in the left frontal cortical region, left basal ganglia, and brainstem (23). These data are partially in line with the earlier results, which pointed to the importance of closeness of the damage focus to the left frontal pole (24), involving prefrontal or basal ganglia structures (25–27), for the PSD appearance.

A resting-state functional MRI study (28) identified changes in the affective network in PSD subjects and revealed that the stroke of the left orbital part of the inferior frontal gyrus was tightly associated with PSD severity. Yang et al. studied the neuroanatomical basis of PSD in relation to white matter connectivity (17). The researchers separated 17 nodes to construct a PSD-related subnetwork. They demonstrated that local efficiency of the subnetwork was significantly declined and this functional decrease was a predictor of PSD (RR:0.84, 95% CI:0.72–0.98). Damages in the left putamen, right insular cortex, and right superior longitudinal fasciculus were found to be correlated with PSD. According to Nys et al., PSD was connected to lesion size but not to lesion location (29).

The discrepancies among studies could be explained by the fact that the relationship between the stroke focus and the likelihood of developing depression may depend on the time since the onset of an acute cerebrovascular accident (30, 31). A large amount of data confirming this point of view has been accumulated. Left-sided stroke lesion was a factor contributing to early onset of PSD (in 2 weeks) (32). Thalamic lesions were significantly associated with PSD in the acute stage of stroke (8). Left lenticulocapsular infarcts were an independent predictor of depressive symptoms at 1 month after stroke onset (33). These data are in agreement with one of the earliest CT-investigations, which revealed the importance of the left hemisphere and basal ganglia lesions for PSD existence at 2 months of the disease (34).

The results of Lassalle-Lagadec et al. study demonstrated that a deterioration of default mode network, which play a key role in mood control, was correlated with PSD severity (35). The investigators revealed that the PSD score at 3-month follow-up was associated with changes in functional connectivity of the left middle temporal cortex and precuneus at 10 days after a first stroke. Ischemic lesions in the frontal-subcortical paths were significantly correlated with depression in contrast to non-depressed subjects at 3 months after stroke onset (36). Moreover, in further logistic regression analysis after adjusting for relevant confounders the relationship (OR:2.6, 95% CI:1.3–5.1) was still significant. Similar data about the crucial role of frontal-subcortical lesions with left-sided predominance (37–39) or without it (40–42) for PSD were also received earlier. Furthermore, Vataja et al. and Lauterbach et al. pointed to the special role of the left pallidum lesions for the onset of PSD (43, 44).

According to a meta-analysis of 31 reports involving 5,309 subjects (18), patients with left hemisphere infarcts might be more vulnerable to PSD during first 6 months after disease onset (OR:1.5, 95% CI:1.2–1.9). Lesions in the left dorsolateral prefrontal cortex are linked to more severe PSD in chronic poststroke patients (45).

It was demonstrated that additional pontine microbleeds in acute ischemic stroke patients significantly increased the possibility of PSD (46), whereas lobar microbleeds decreased the remission rate of PSD (47). Tang et al. also received results, which suggested that lobar microbleeds might be crucial for the PSD onset in subjects with lacunar infarcts (48).

The results of some studies revealed that the type of ischemic stroke might be a risk factor for PSD. Subjects with total anterior cerebral infarcts had a greater prevalence of depression (OR:1.76, 95% CI:1.1–2.7) (49). At the same time, Arba et al., exploring the association between different subcortical ischemic stroke lesions and occurrence of PSD at 1 year after disease onset, established that the lacunar subtype was least associated with PSD (OR:0.71, 95% CI:0.55–0.93) compared to other stroke variants (50). Nevertheless, accumulation of subcortical lacunar lesions in the deep white matter, basal ganglia, and thalamus might be a more significant predictor of PSD than solitary lacunas (51, 52).

In some studies, the influence of different types of cerebral vasculopathy for PSD in ischemic stroke patients was assessed. Enlarged perivascular spaces (markers of cerebral small vessel disease) in the centrum semiovale on axial T2 weighted MRI independently predicted PSD occurrence at a 3-month period after stroke, according to the Liang et al. data (53). Severe white matter hyperintensities, another cerebral sign that reflects cerebral microvasculopathy, were identified as an independent factor of PSD at 3 months after stroke onset (54). Moreover, intracranial atherosclerosis on MRI scans might be essential for prediction of PSD in ischemic stroke subjects (55). In general, mentioned data are in accordance with the meta-analytical evidence that the reduction of overall brain perfusion has an impact on PSD (56).

Few studies have examined clinical and neuroimaging correlations of PSD after hemorrhagic stroke. Stern-Nezer et al. after investigating 89 patients with spontaneous intracerebral hemorrhage concluded that PSD was not associated with hematoma volumes and presence of intraventricular hemorrhage (57).

Summarizing obtained neuroimaging data in PSD patients, the conclusion can be made that clinical-anatomical correlations were found only for ischemic stroke lesions. Localization of the focus, the volume of ischemia, and additional burdening anatomical factors were contributing to PSD occurrence. For 1 year after the stroke onset, lesions affecting the frontal-subcortical affective network (prefrontal cortex, basal nuclei, and thalamus) predominantly in the left hemisphere can be considered as neuroimaging markers for PSD and as predictors of PSD development. Total anterior cerebral infarcts lead to a higher PSD occurrence, whereas lacunar lesions less often cause depression symptoms. Supplementary pontine and lobar cerebral microbleeds in acute stroke patients, as well as severe brain microvasculopathy, increase the likelihood of PSD.

## Molecular biomarkers of PSD

### Monoamines

Molecular markers of PSD are presented in Table 2. It was revealed that PSD patients had significantly lower liquor concentrations of 5-hydroxyindoleacetic acid (a 5-HT metabolite) compared to non-depressed subjects with acute stroke lesions and non-depressed patients without stroke lesions (92). The results demonstrate that serotonergic mechanisms are implicated in PSD pathogenesis. Nevertheless, these data need to be further confirmed.

**Table 2:** Summary of molecular biomarkers of PSD.

**Study**	**Type of study**	**Patients**	**N**	**Depression instrument**	**Results**
**GROWTH FACTORS**					
Noonan et al. (56)	Meta-analysis	Poststroke	1893	DSM, 9 different standardized mood scales	Moderate effect for lower serum BDNF levels (SMD: −0.52, 95% CI: −0.84 to −0.21, *P* = 0.001) to poststroke depression
Xu et al. (58)	Meta-analysis	PSD Non-PSD	171 328	HDRS, DSM-III-R, DSM-IV, DSM-IV	In the acute stage of stroke, serum BDNF levels were lower in PSD patients compared with non-PSD (SMD: −1.43, 95% CI:−2.56 to −0.31, *P* = 0.01)
**MARKERS OF NEUROINFLAMMATION**					
Tang et al. (59)	Cross-sectional	PSD Non-PSD	69 157	DSM-IV, HDRS	Significantly higher Hs-CRP levels were found in PSD patients compared to controls (1.54 (1.04–2.25) vs. 0.41 (0.20–1.13) mg/dL, *P* < 0.0001). After adjusting all other possible covariates, Hs-CRP levels were independent predictors of PSD at 6 months after stroke (OR:1.33, 95% CI:1.23–1.45, *P* < 0.001) with AUC of 0.77 (95% CI, 0.70–0.98)
Yang et al. (60)	Cross-sectional	PSD Non-PSD	69 157	DSM-IV, HDRS	At admission, significantly higher Hs-CRP levels in PSD group than in non-PSD (1.54 (0.79–2.27) vs. 0.43 (0.31–1.27) mg/dL, *P* < 0.0001). After adjusting for confounders, Hs-CRP still was an independent predictor of PSD (OR:1.34, 95% CI:1.23–1.46, *P* < 0.001). An increased risk of PSD was associated with serum Hs-CRP levels ≥ 0.85 mg/dL (OR:7.83, 95% CI:4.19–14.62) after adjusting for confounders
Zhu et al. (61)	Prospective	PSD Non-PSD	56 140	DSM-IV, HDRS	In multivariate analyses, serum levels of ferritin ≥130.15 μg/L were independently associated with PSD at 2 months (OR:5.39, 95% CI:1.73–16.83; *P* = 0.004) after adjusting for all variables
Tang et al. (62)	Prospective	PSD Non-PSD	69 157	DSM-IV, HDRS	At admission, PSD patients compared to non-PSD showed higher levels of serum neopterin (21.6 (18.9–25.7) vs. 14.6 (12.2–18.4) nmol/L, *P* < 0.0001). In multivariate analyses, serum neopterin was an independent predictor of PSD at 6 months (OR:1.95, 95% CI:1.36–2.81, *P* < 0.0001). With an AUC of 0.85 (95% CI, 0.80–0.90), neopterin showed a significantly great discriminatory ability
Cheng et al. (63)	Prospective	PSD Non-PSD	70 139	DSM-IV, HDRS	PSD patients compared with non-PSD had higher levels of plasma glutamate (299 (235–353) vs. 157 (108–206) μM, *P* < 0.0001). In multivariate analyses, plasma glutamate level was an independent predictor of PSD at 3 months (OR:1.03, 95% CI:1.02–1.04, *P* < 0.0001). Plasma glutamate levels >205 μM were independently associated with PSD (OR:21.3; 95% CI:8.28–67.36, *P* < 0.0001)
Su et al. (64)	Prospective	PSD Non-PSD	12 92	DSM-IV, HDRS	Proinflammatory cytokines were significantly elevated in PSD patients compared to non-PSD: IL-6 (32.84 ± 71.51 vs. 1.47 ± 4.28, *P* < 0.001), IL-10 (15.02 ± 31.34 vs. 5.01 ± 8.77, *P* = 0.015), TNF-α (16.90 ± 28.54 vs. 2.08 ± 3.48 *P* < 0.001), and IFN-γ (8.16 ± 11.56 vs. 0.21 ± 1.28 *P* < 0.001). PSD patients had significantly elevated serum ratios of IL-6/IL-10 (z = −3.06, *P* = 0.002) and TNF-α/IL-10 (z = −2.23, *P* = 0.03), but not in IL-1β/IL-10 (z = −0.29, *P* = 0.8) and IFN-γ/IL-10 (z = −1.14, *P* = 0.3). A little difference in IL-1β between groups was found as the concentration was too low to be detected
Bensimon et al. (65)	Cross-sectional	PSD Non-PSD	30 56	CES-D	Middle CES-D tertile scores were correlated with higher serum IL-1β level [*F*_(2, 52)_ = 3.55, *P* = 0.04] and serum ratios of IL-18/IL-10 [*F*_(2, 52)_ = 3.30, *P* = 0.046], IFNγ/IL-10 [*F*_(2, 52)_ = 4.02, *P* = 0.025], IL-1β/IL-10 [*F*_(2, 52)_ = 4.34, *P* = 0.02]
Swardfager et al. (66)	Cross-sectional	PSD Non-PSD	19 28	CES-D	IL-17 concentrations did not differ between subjects with and without depressive symptoms [*F*_(1, 46)_ = 8.44, *P* = 0.57); IL-17 was associated with poorer cognitive status in PSD subjects [*F*_(1, 46)_ = 9.29, *P* = 0.004]. In those subjects, IL-17 was associated with higher lipid hydroperoxide (ρ = 0.518, *P* = 0.02) and lower IL-10 (ρ = −0.484, *P* = 0.04), but not in those without.
Nguyen et al. (67)	Cross-sectional	Poststroke	44	MADRS	Two innate immune pathways in peripheral bloods, complement and coagulation, trend toward downregulation at the 3-month phase in correlation with mild symptoms of PSD
Han et al. (68)	Cross-sectional	PSD Non-PSD HC	55 134 100	DSM-IV, HAM-D	Serum vitamin D levels within 24 h after admission were significantly lower in non-PSD and PSD patients than in HC (*P* < 0.05). Serum vitamin D levels (≤ 37.1 and ≥64.1 nmol/L) were independently associated with the development of PSD (OR:8.82, 95% CI:2.01–38.72, *P* = 0.004, and OR:0.13, 95% CI:0.02–0.72, *P* = 0.02, respectively)
**MARKERS OF HYPOTHALAMIC-PITUITARY-ADRENAL AXIS**					
Mitchell (69)	Review	n/a	n/a	n/a	In the medium term (1 month to 1 year) hypercortisolemia is related to the development of a PSD and relates to functional outcome and survival
Kwon et al. (70)	Case-control	PSD HC	28 23	BDI, HDRS	Cortisol level of PSD patients did not rise significantly at any sampling time, showing a flat curve. The difference between cortisol levels of PSD and HC was significant at all sampling time points except for immediate postawakening (*t* = 4.15, *P* < 0.001; *t* = 4.21, *P* < 0.001; *t* = 2.33, *P* < 0.03, for 15, 30, and 45 min after awakening, respectively). Cortisol response after awakening correlated significantly with both the BDI and HDRS scores (for BDI, *r* = −0.75, *P* < 0.001; for HDRS, *r* = −0.71, *P* < 0.001)
Noonan et al. (56)	Meta-analysis	Poststroke	1893	DSM, 9 mood scales	Moderate effects for high postdexamethasone cortisol levels were detected (OR:3.28; 95% CI 1.28–8.39, *P* = 0.01) to poststroke depression
Harney et al. (71)	Cross-sectional	Poststroke	12	HDRS	At 1 week poststroke, DST results were abnormal in 75% of the patients; at 3 weeks poststroke DST results were abnormal in 50% of the patients
Lipsey et al. (72)	Prospective	Poststroke	48	ZSDS	DST sensitivity was only 67%, the specificity was only 70%, and diagnostic confidence was only 50%
Grober et al. (73)	Cross-sectional	Poststroke	29	n/a	DST's sensitivity was 15%, its specificity was 67%, and its positive predictive value was 48%. DST yields no more information than would be gained from random assignment of the diagnosis of depression
**METABOLITES**					
Ormstad et al. (74)	Prospective	PSD Non-PSD	45	BDI	BDI score at 12 months poststroke was positively correlated with the acute serum level of glucose (*r* = 0.32, *P* = 0.04). High acute serum levels of glucose (≥ 126 mg/dL) may predict PSD after acute ischemic stroke
Gu et al. (75)	Prospective	PSD Non-PSD	56 140	DSM-IV, HDRS-17	PSD patients as compared to non-PSD patients showed significantly lower levels of uric acid at baseline (237.02 ± 43.43 vs. 309.10 ± 67.44 mmol/L, *P* < 0.001). In multivariate analyses, uric acid levels (≤ 239.0 and ≥ 328.1 mmol/L) were independently associated with the development of PSD (OR:7.76, 95% CI:2.56–23.47, *P* < 0.001 and OR:0.05, 95% CI:0.01–0.43, *P* = 0.01)
Tang et al. (76)	Prospective	PSD Non-PSD	61 574	DSM-IV	Significant differences were found between the PSD and non-PSD groups in terms of bilirubin level (*P* = 0.006). In *post-hoc* comparisons, the proportion of patients with bilirubin ≥14.1 mmol/L was significantly higher in the PSD group (37.7 vs. 19.7%, *P* = 0.001). In the final regression model, bilirubin level ≥14.1 mmol/L remained a significant independent predictor of PSD (OR:2.4)
Zhang et al. (77)	Case-control	PSD Non-PSD HC	28 39 40	HDRS	Expression of ApoE mRNA was significantly lower in mononuclear blood cells in PSD group than in non-PSD (0.77 ± 0.24 vs. 0.86 ± 0.14, *t* = 2.85, *P* = 0.006) and serum ApoE was significantly higher in the PSD group than in non-PSD (0.99 ± 0.23 vs. 0.85 ± 0.28 mg/dL, *t* = 2.01, *P* = 0.048)
Lee et al. (78)	Case-control	PSD Non-PSD	60 70	DSM-IV, BDI	Higher serum leptin levels were found in PSD group compared to non-PSD (38.5 (25.1–59.2) vs. 8.2 (4.9–17.8) ng/mL, *P* < 0.01)
Jiménez et al. (79)	Prospective	PSD Non-PSD	109 25	DSM-IV, GDS	PSD patients compared to non-PSD had higher serum leptin levels at discharge (43.4 (23.4–60.2) vs. 6.4 (3.7–16.8) ng/ml, *P* < 0.001) and at 1 month after stroke (46.2 (34.0–117.7) vs 6.4 (3.4–12.2) ng/ml, *P* < 0.001)
Xiao et al. (80)	Case-control	PSD Non-PSD HC	62 45 43	DSM-IV, HDRS	A panel of five metabolites—lactate, α-hydroxybutyrate, phenylalanine, formate, and arabinitol – could yield a diagnostic accuracy of 83.9% in the training set and a predictive accuracy of 81.3% in the testing set for PSD diagnosis. The AUC value of this panel was 0.95 (95% CI:0.91–0.98, *P* = 0.0001) in the training set and 0.95(95% CI: 0.91–0.99, *P* = 0.0001) in the testing set
Zhang et al. (81)	Case-control	PSD Non-PSD HC	130 128 127	DSM-IV, HDRS-17	Azelaic acid, glyceric acid, pseudouridine, 5-hydroxyhexanoic acid, tyrosine, and phenylalanine could effectively discriminated between PSD subjects and non-PSD subjects, achieving AUC of 0.96 in a training set and this urinary biomarker panel was capable of discriminating blinded test samples with an AUC of 0.95
**GENETIC MARKERS**					
Kohen et al. (82)	Case-control	PSD Non-PSD	75 75	GDS-30	Individuals with the 5-HTTLPR s/s genotype had higher risk of PSD compared with l/l or l/xl genotype carriers (OR:3.1, 95% CI:1.2–8.3). Participants with the STin2 9/12 or 12/12 genotype had 4-fold higher odds of PSD compared with STin2 10/10 genotype carriers (OR:4.1, 95% CI:1.2–13.6)
Ramasubbu et al. (83)	Case-control	PSD Non-PSD	26 25	DSM-IV	The homozygosity of short alleles was significantly associated with poststroke major depression (χ^2^ = 6.04, df = 1, *P* = 0.025)
Ramasubbu et al. (84)	Case-control	PSD Non-PSD	26 25	DSM-IV	There were significant intergroup differences in the allelic frequencies of 5-HTTLPR/rs25531 (S_A_, L_A_, and L_G_) (*P* < 0.05) and in the combined frequencies of lower expressing alleles (S_A_ and L_G_) and higher-expressing alleles (L_A_) (*P* = 0.025) between subjects with PSD and non-PSD
Fang et al. (85)	Case-control	PSD Non-PSD	57 57	DSM-IV, HDRS	S/S genotype was significantly higher *(P* = 0.049) in the PSD group than in the control group
Kim et al. (86)	Cross-sectional	PSD Non-PSD	77 199	DSM-IV, MINI	5-HTR2a 1438 A/A genotype was associated with major PSD, while 5-HTTLPR s/s and BDNF met/met genotypes were associated with all PSD. There was a significant interaction between 5-HTR2a 1438A/G and BDNF val66met polymorphisms for major PSD and a borderline significant interaction between 5-HTTLPR and BDNF val66met polymorphisms for all PSD
Guo et al. (87)	Cross-sectional	PSD Non-PSD	199 202	DSM-IV	Significant differences were found in genotype (χ^2^ = 16.75, *P* = 0.002) and allele (χ^2^ = 15.12, *P* = 0.001) frequencies between the groups. SS genotype distribution of 5-HTTLPR and S allele frequencies in PSD patients are higher than in non-PSD patients. The frequency of the L allele was significantly lower in PSD patients than in non-PSD patients (OR:0.53, 95% CI = 0.39–0.72)
Kim et al. (88)	Longitudinal	Poststroke	222	HADS, HDRS	Higher SLC6A4 promoter methylation status was independently associated with PSD both at 2 weeks and more prominently at 1 year after stroke, and was significantly associated with the worsening of depressive symptoms over 1 year. These findings were significant only in the presence of the 5-HTTLPR S/S genotype
Lee et al. (89)	*Post-hoc* analysis	Poststroke	301	MADRS	Among escitalopram users (*n* = 159), neurological function in subjects with STin2 12/10 (*n* = 29) improved significantly more than that in STin2 12/12 carriers (*n* = 130). After adjusting, STin2 12/10 independently predicted a good clinical outcome at 3 months (OR:2.99, 95% CI:1.04–8.58)
Tang et al. (90)	Cross-sectional	PSD Non-PSD	61 162	GDS-15	There were significant associations between the HTR2C gene and PSD status in male patients, but not in female. After adjusting for possible confounders, rs12837651 T allele (OR:4.02, 95% CI:1.16–13.93) and rs2192371 G allele (OR:2.87, 95% CI:1.06–7.75) were significantly associated with PSD in males
Kim et al. (91)	Longitudinal	Poststroke	222	HADS, HDRS	Higher BDNF methylation status was independently associated with prevalent, persistent and particularly with incident PSD, and with worsening depressive symptoms over follow-up

*BDI, Beck Depression Inventory; CES-D Center for Epidemiological Studies Depression scale; CI, confidence interval; DSM, Diagnostic and Statistical Manual; GDS, Geriatric Depression Scale; HADS, Hospital Anxiety and Depression Scale; HC, healthy controls; HDRS, Hamilton Depression Rating Scale; IQR, interquartile range; MADRS, Montgomery and Asberg Depression Rating Scale; MINI, Mini-International Neuropsychiatric Interview; Non-PSD, poststroke patients without depression; OR, odds ratio; PSD, poststroke patients with depression; SMD, standardized mean difference; ZSDS, Zung Self Report Depression Scale*.

### Growth factors

Accumulating evidence shows that expression of brain-derived neurotrophic factor (BDNF) is involved in the pathophysiological mechanisms of depression (93) and PSD (94). A meta-analysis of four studies including 499 stroke patients (58) revealed that significant decrease in serum BDNF concentrations in the early period after stroke predisposed to the development of depression. The data correspond to the results of an earlier meta-analysis Noonan et al. (56).

A growing amount of evidence indicates the pathogenic influence of insulin-like growth factor 1 (IGF-1) on a major depressive disorder (MDD) (95). Most of the studies showed that increased peripheral IGF-1 levels might predict the occurrence of MDD, whereas decreased IGF-1 levels might reflect the treatment effectiveness (96). Nevertheless, Yue et al. found no differences in serum IGF-1 concentrations in PSD patients as opposed to non-depressed poststroke patients and persons with MDD (97). On the other hand, the authors revealed significantly greater serum IGF-1 mRNA concentrations in PSD group compared to depressed subjects without stroke (97).

Therefore, the decrease of serum BDNF concentrations after stroke can be used as a PSD predictor.

### Markers of neuroinflammation

Despite serious methodological issues, current research found that immune dysfunction is crucial for the pathophysiology of PSD (98). Immunological mechanisms can initiate the inflammation-bound cell death in mood-related cerebral areas (99). Therefore, markers of neuroinflammation could be helpful for PSD diagnosing.

It was shown that increased early markers of inflammation predicted further PSD development. Tang et al. found that elevated serum concentrations of high-sensitivity C-reactive protein (Hs-CRP) in the acute stroke phase independently predisposed to PSD occurrence at 6 months after its onset (OR:1.3, 95% CI:1.2–1.5, and AUC value of 0.765, 95% CI:0.701–0.983) (59). Similar results were published by Yang et al. (60). The authors also established that higher risk of PSD is related to serum Hs-CRP concentrations ≥ 0.85 mg/dL (OR:7.8, 95% CI:4.2–14.6) (60).

Serum level of ferritin (an inflammatory acute phase protein) ≥ 130.15 μg/L was independently related to depression after a 2-month period of stroke onset (OR:5.4, 95% CI:1.7–16.8) in accordance with Zhu et al. (61). Patients with PSD showed higher levels of serum neopterin (a marker of cellular immune system activation) at admission compared with non-depressive poststroke subjects (21.6 vs. 14.6 nmol/L) (62). Furthermore, serum neopterin independently predicted PSD occurrence after 6 months of stroke onset (OR:1.95, 95% CI:1.36–2.81) and demonstrated a prominently higher discriminatory ability when compared to Hs-CRP.

Increased plasma glutamate concentrations (>205 μM) at early stage predicted PSD development at 3 months after stroke (OR:21.3, 95% CI:8.3–67.4) (63). It was suggested that glutamate has an inflammatory potential due to its ability to initiate immunological processes in the nervous system (100).

Particular attention has been paid to cytokine-related markers of PSD. Su et al. found significant increases of several serum cytokines [tumor necrosis factor α (TNF-α), interleukin-6 (IL-6), interferon-γ (IFNγ)], as well as in the pro-inflammatory/anti-inflammatory ratios of TNF-α/IL-10 and IL-6/IL-10 in PSD subjects after 1, 3, 6, 9, and 12 months of stroke (64). Interleukin-1β was too low to show any difference (64). Moreover, Bensimon et al. showed that serum concentrations of pro-inflammatory cytokine IL-1β and serum pro-inflammatory/anti-inflammatory ratios of IL-18/IL-10, IFNγ/IL-10, and IL-1β/IL-10 were increased in patients with moderate severity of PSD (65). At the same time, peripheral kynurenine/tryptophan ratios, which had been earlier suggested to connect neuroinflammatory, neurotoxical, and neurotransmitter processes, were not associated with PSD (65).

Interestingly, IL-17 serum levels did not distinguish poststroke patients with and without PSD in Swardfager et al. study (66). Nevertheless, IL-17 was correlated with lower cognitive functioning in PSD patients. In those depressive individuals, IL-17 was related to increased lipid hydroperoxide, a measure of oxidative stress (ρ = 0.52), and decreased IL-10 (ρ = −0.48), in contrast to subjects without PSD (66). Authors concluded that cognitive PSD symptoms might be linked to IL-17 related signaling, including pro-inflammatory and anti-inflammatory imbalance and hyperoxidation.

Cytokines can realize their pathogenic impact by driving intrinsic apoptotic pathways, involving intracellular calcium, glutamate excitotoxicity, and reactive oxygen species, which significantly elevates the risk of depression (99). It was also hypothesized that the increased production of pro-inflammatory cytokines due to stroke may amplify the pro-inflammatory processes, predominantly in limbic regions, extensively activate indoleamine 2, 3-dioxygenase, and, consequently, reduce serotonin production in paralimbic areas, including ventral lateral frontal and polar temporal cortex, as well as basal ganglia. The sequential physiological dysregulation might result in PSD (101).

The complement system is usually considered to be an essential part of the innate immunity and a linkage to the acquired immunity throughout pro-inflammatory cytokine transmission. Nguyen et al. revealed that lowered complement expression in serum was associated with PSD symptoms at 3 months after stroke (67).

Some studies revealed that decreased concentrations of vitamin D, which is essential for immunoregulation, were associated with depression in poststroke patients (68, 102). PSD subjects had significantly decreased serum concentrations of vitamin D within 24 h after admission compared to non-depressed individuals. Serum vitamin D lower than 37.1 nmol/L were independently related to the onset of PSD (OR:8.8, 95% CI:2.0–38.7) (68).

Thus, increased early markers of inflammation (Hs-CRP, ferritin, neopterin, and glutamate), in addition to elevated serum pro-inflammatory cytokines (TNF-α, IL-1β, IL-6, IL-18, IFN-γ) and pro-inflammatory/anti-inflammatory ratios (TNF-α/IL-10, IL-1β/IL-10, IL-6/IL-10, IL-18/IL-10, IFN-γ/IL-10) might be used for underpin of PSD diagnosis. PSD is also characterized by lowered complement expression and decreased serum vitamin D levels. The relationship between IL-17 levels and cognitive functioning in PSD patients need to be replicated in further investigations.

### Markers of hypothalamic-pituitary-adrenal axis (HPA)

According to review (69), persistent HPA dysregulation occurs in up to 40% of stroke patients. The level of hypercortisolemia is moderately determined by the volume and location of the infarct. In 1-month to 1-year term after stroke onset hypercortisolemia is correlated with PSD (69). Kwon et al. compared the cortisol awakening response (measuring saliva cortisol directly, 15, 30, and 45 min after wakening) in PSD patients (2 months after a stroke or longer) with age-matched controls (70). In PSD group, salivary cortisol concentrations did not increase considerably at any measured time, demonstrating the blunted cortisol awakening response. Furthermore, a prominent adverse association between the cortisol awakening response and the severity of depression in PSD group was shown (70).

It was detected in the meta-analysis Noonan et al. that dexamethasone moderately suppressed elevated cortisol concentrations (OR:3.3, 95% CI:1.3–8.4) in PSD patients (56). Abnormal dexamethasone suppression test (DST) results at 3 weeks after stroke might be a potential PSD predictor (71). Nevertheless, some investigators declared low utility of the test. Thus, Lipsey et al. found that DST sensitivity and specificity was only 67 and 70%, respectively (72). Grober et al. indicated that the DST sensitivity and specificity was only 15 and 67%, respectively, whereas, the positive predictive value was 48% (73). Therefore, it was concluded that the DST provides no more information for PSD diagnosis (73).

Thereafter, it can be suggested that among HPA axis dysfunction markers, hypercortisolemia and the blunted cortisol awakening response are the most prominent in PSD patients.

### Markers of oxidative damage

A great amount of evidence suggests that stroke is accompanied by oxidative stress. Some studies investigated the links between oxidative stress and PSD. Cichon et al. evaluated a possible relationship between plasma protein oxidative damage and the likelihood of PSD (103). The research showed that oxidative proteins damage was associated with the severity of PSD. Liu et al. found a positive correlation between serum malondialdehyde (oxidative stress biomarker) levels and PSD severity (*r* = 0.54) during 1 month follow up after stroke onset (104). According to the ROC-analysis, the optimal cutoff value of serum malondialdehyde concentrations as an indicator to support a PSD diagnosis was 2.898 nmol/mL, which yielded a sensitivity and a specificity of 77.9 and 81.1%, respectively, with AUC of 0.883 (95% CI:0.836–0.929). Elevated malondialdehyde higher than 2.898 nmol/mL was an independent predictive marker of PSD (OR:24.3, 95% CI:9.5–62.4).

Nevertheless, specificity of mentioned oxidative markers for delineating PSD and non-PSD poststroke patients should be supported in larger sample studies. Therefore, they couldn't be recommended for routine clinical practice.

### Metabolites

Evidence suggests that elevated acute serum glucose concentrations might be a predictor of PSD after ischemic stroke. The PSD score (according to Beck Depression Inventory) at 12 months after a stroke had a positive association with the serum glucose concentration at admission (*r* = 0.32) (74). The authors established that the acute glucose concentrations higher than 126 mg/dL could be a predictor of PSD occurrence.

Gu et al. examined a possible association between serum uric acid levels within 24 h after stroke onset and the development of PSD at a 3-month poststroke period (75). They demonstrated that uric acid concentrations lower than 239.0 and higher than 328.1 μmol/L were independently related to the onset of PSD (OR:7.76, 95% CI:2.56–23.47, and OR:0.05, 95% CI:0.01–0.43, respectively). Summarizing previously obtained data, authors concluded that possible antidepressant effects of uric acid could be explained by its multiple antioxidant (scavenging of free radicals and reactive oxygen species, chelation of transition metals, prevention of lipid peroxidation) and anti-inflammatory roles, decreasing blood-brain barrier permeability, and, consequently, diminishing central nervous tissue damage and neuronal death (75).

Substantial differences were established between the PSD and non-PSD patients with acute stroke regarding bilirubin concentrations (76). In *post-hoc* comparisons, the percentage of subjects with bilirubin ≥ 14.1 μmol/L was significantly greater among PSD patients (37.7 vs. 19.7%). After the final regression analysis, bilirubin concentration ≥ 14.1 μmol/L still independently predicted PSD. According to the authors‘ view, high bilirubin concentrations in PSD patients may reflect the intensity of initial oxidative stress, as well as indicate a higher level of perceived psychological stress (76).

ApoE plays a key role in lipid metabolism regulation. It was indicated that PSD individuals are more likely to demonstrate dyslipidemia and abnormal serum ApoE levels (105). Zhang et al. established that compared to non-depressed poststroke patients, PSD subjects had decreased peripheral ApoE microRNA expression and increased serum ApoE (77). Higher serum leptin (a hormone predominantly made by adipose cells) concentrations were found in PSD group in comparison with non-PSD poststroke individuals (38.5 vs. 8.2 ng/mL) (78). Increased serum levels of leptin at 7th and 30th day of poststroke also showed a correlation with later onset of PSD (79).

Xiao et al. used nuclear magnetic resonance spectroscopy-based metabonomic analysis to determine urine metabolites that are significantly altered in PSD patients (80). This approach could differentiate PSD patients from healthy control and non-depressed poststroke patients with high accuracy. Authors identified the panel of urinary metabolites, which included arabinitol, formate, lactate, phenylalanine, and α-hydroxybutyrate. They found that PSD patients had higher urine concentrations of lactate and α-hydroxybutyrate and lower urine concentrations of phenylalanine, formate, and arabinitol compared to healthy controls. The satisfactory predictability of the panel (AUC of 0.946) demonstrated that it could be a “good” classifier for PSD diagnosing (80).

Zhang and Zhang (81) proposed a combined panel of six urinary biomarkers (azelaic acid, glyceric acid, phenylalanine, pseudouridine, tyrosine, and 5-hydroxyhexanoic acid) that might separate PSD patients from non-depressed poststroke individuals with AUC of 0.961 in a training set and 0.954 in discriminating blinded test samples.

Overall, considerable amount of limitations including restricted ethnical groups, a single metabolomics platform, inability to differentiate PSD from stroke patients with other neuropsychiatric disorders, and the necessity to collect cerebrospinal fluid from PSD patients to ensure that mentioned above serum/plasma/urinary markers are relevant to the focus of disease pathogenesis require future multinational investigations with appropriate methodology.

### Genetic markers

A number of studies consider that PSD might be caused by genetic susceptibility. Much attention has been paid to the serotonin transporter gene SLC6A4 polymorphisms, especially 5-HTTLPR, STin2 VNTR, and rs25531. Summarizing data about them were presented in Kohen et al. review (82). The SERT gene is found on chromosome 17q11.1-17q12 and includes 14 exons. SERT most often studied variant 5-HTTLPR, which is found in the promoter region, is divided into a long (L) and short (S) allele, based on the absence or presence of a 43 bp deletion/insertion polymorphism. rs25531 is a single nucleotide polymorphism (SNP), existing in a common (A) or rare (G) variant, which location is immediately upstream of 5-HTTLPR in the SERT gene. A STin2 VNTR SERT polymorphism is situated in intron 2 and comprises a variable number of nearly equal 17 bp segments (usually 9, 10, or 12).

Several studies were devoted to the serotonin transporter gene-linked polymorphic region (5-HTTLPR) genotype in PSD subjects. Most studies showed that S/S (short allele) 5-HTTLPR genotype was significantly more frequent in PSD patients compared to non-depressed poststroke subjects (82–87). In contrast, LL (long allele) genotype was more prevalent in non-depressed poststroke subjects compared to PSD patients (87). This regularity can be explained by the fact that the transcription capacity of the S allele is lower to that of the L allele, leading to poor serotonin expression in the areas of action (106).

In addition to the 5-HTTLPR polymorphism, expression of SLC6A4 is influenced by DNA methylation status. Kim et al. found that hypermethylation of SLC6A4 promoter was independently related to PSD at 2 weeks and 1 year after ischemic brain incident. Moreover, it was significantly linked to the increase of depression severity during a 1-year poststroke period (88).

Stroke patients with the STin2 9/12 or 12/12 genotype showed a 4-fold elevated risk of PSD occurrence than individuals with STin2 10/10 genotype (OR:4.1, 95% CI:1.2–13.6) (82). It was also shown that serotonin transporter intron 2 (STin2) 12/10 variable number tandem repeat genotype might be associated with a good clinical outcome in PSD patients after 3 months of selective serotonin reuptake inhibitors (escitalopram) therapy (89). An association of rs25531 with PSD was not established (82).

Few studies assessed the association between some serotonin receptors (5-HTR) and growth factors genotypes and PSD. 5-HTR2a (serotonin 2a receptor) 1438 A/A genotype was linked to major PSD, whereas BDNF met/met genotype was linked to major as well as minor PSD (86). The authors found a substantial association between 5-HTR2a 1438A/G and BDNF val66met polymorphisms for major PSD and a marginally significant association between BDNF val66met polymorphisms for both (major and minor) PSD (86). Furthermore, Tang et al. established considerable interactions between the HTR2c gene and PSD presence in the male Chinese poststroke individuals (90). They showed that rs12837651 T allele and rs2192371 G allele significantly correlated to PSD status with OR of 4.02, 95% CI:1.16–13.93, and 2.87, 95% CI:1.06–7.75, respectively (90).

Along with genetic profiles, BDNF secretion is influenced by epigenetic factors. In this regard, Kim et al. (91) demonstrated that BDNF promoter hypermethylation independently correlated with the prevalence, persistence, and incidence of PSD, as well as with aggravating of depression severity over a 1-year period after stroke (91).

A potential role of microRNAs in PSD pathogenesis was observed in the review of experimental studies by Yan et al. (107). Implementation of those results into clinical practice may be helpful for the diagnosis and prognosis of PSD.

On the whole, genetic markers, namely S/S 5-HTTLPR, STin2 9/12, and 12/12 genotypes of the serotonin transporter gene SLC6A4, 5-HTR2a 1438 A/A, and BDNF met/met genotypes, can reflect the hereditary predisposition of PSD. To epigenetic factors of PSD, higher SLC6A4 promoter and BDNF promoter methylation status can be referred.

## Neurophysiological markers

### EEG

To assess abnormalities in EEG complexity in PSD subjects Zhang et al. used Lempel-Ziv Complexity (108). It was shown that PSD individuals had lower neural complexity in whole cerebral areas compared with poststroke non-depressed persons and healthy controls. As screening indicators for PSD, Lempel-Ziv Complexity parameters demonstrated more than 85% in specificity, sensitivity, and accuracy (Table 3). The lack of severe PSD patients in this study and absence of correlations between a stroke location and neurophysiological data do not allow expanding obtained data on the entire PSD population currently.

**Table 3:** Summary of neurophysiological markers of PSD.

**Study**	**Type of study**	**Patients**	***N***	**Depression instrument**	**Results**
Zhang et al. (108)	Case-control	PSD Non-PSD HC	21 22 15	DSM-IV, HDRS-24	PSD patients showed lower neural complexity compared with non-PSD and HC in the whole brain regions. In stroke patients, significant correlation was between HDRS and LZC in the whole brain regions, especially in frontal and temporal. LZC parameters used for PSD recognition possessed more than 85% in specificity, sensitivity and accuracy
Wang et al. (109)	Case-control	PSD Non-PSD	26 32	HDRS	For stroke subjects with left-side lesions, only increased beta2 power in frontal (F3 and F4) and left central (C4) areas was found in PSD patients. Stroke subjects with right-side lesions showed increased theta power in occipital (O1 and O2) and frontal-temporal (F7, T4, T5, and T6) areas, as well as increased alpha power in left occipital (O1) and temporal areas (T4, T5, and T6). Beta2 power in central regions and parietal regions of the right hemisphere showed strong discrimination of PSD with left hemispheric lesion, for which the AUC values reached 0.8. Theta power showed strong discrimination of PSD with right hemispheric lesion in most of the right hemisphere (F4, C4, P4, O2, T4, T6) and in prefrontal and temporal regions of the left hemisphere (FP1, F7, T3, T5), for which the AUC values reached 0.8. No significant association was found between any of the frequency power and depressive severity in PSD subjects with lesions in the right or left hemisphere
Zhang et al. (77)	Case-control	PSD Non-PSD HC	28 39 40	HDRS	Poststimulus latencies of the N200 and P300 ERP components were significantly prolonged in patients with PSD compared to non-PSD and HC (FN200 = 152.52, *P* = 0.008; FP300 = 51.19, *P* = 0.002), while the P300 amplitude was significantly reduced in PSD compared to other groups (FP300 = 40.63, *P* = 0.005)
Wenzhen et al. (110)	Case-control	PSD Non-PSD	85 85	HDRS	The N400 incubation periods and averaged amplitudes of the PSD patients were statistically different from those of the control (*t* = 15.79, *P* < 0.01 and *t* = 11.22, *P* < 0.01, respectively)

*DSM, Diagnostic and Statistical Manual; HC, healthy controls; HDRS, Hamilton Depression Rating Scale; LZC, Lempel-Ziv Complexity; Non-PSD, poststroke patients without depression; PSD, poststroke patients with depression*.

Wang et al. examined quantitative EEG changes in PSD subjects with basal ganglia infarcts (109). Left-hemisphere PSD patients showed increased beta2 power in frontal and central areas, whereas right-hemisphere PSD ones showed increased theta and alpha power mainly in occipital and temporal regions. Additionally, for left-hemisphere lesions, beta2 power in central and right parietal regions provided high discrimination between PSD and poststroke non-depressed subjects, and for right-hemisphere lesions, theta power was similarly discriminative in most regions, especially in temporal regions. No relationship was found between the symptoms of depression and the power of abnormal bands. Small sample size of patients including basal ganglia lesions selectively do not provide an opportunity to recommend the methodology without replication in further studies.

Zhang et al. assessed event-related potentials (ERPs) in PSD patients (77). The average incubation period of N200 and P300 ERPs waves was prolonged, and the P300 amplitude was decreased in PSD subjects in comparison with non-PSD stroke patients and healthy individuals (*P* < 0.01). Wenzhen et al. also revealed that the incubation period of N400 was significantly prolonged and the average amplitude of the ERPs component was reduced in PSD group in comparison with those in the non-PSD group (110). These findings demonstrate that PSD subjects are prominently worse at recognizing target stimuli, indicating lower perceptual abilities and/or cognitive processing (77, 110). Therefore, neurophysiological markers of PSD, reflecting a violation of perception and cognitive processing, are the elongation of the latency of N200, P300, and N400, as well as a decrease in the P300 and N400 amplitude of the ERPs, could be used in the diagnosis process.

## Conclusion

Summarizing obtained data, we can highlight that revealed biomarkers reflect complicated neurobiological mechanisms of PSD (111). They are caused by neuroanatomical substrates and involve different molecular signal pathways including serotonergic dysfunction, growth factors failure, neuroinflammation, HPA dysregulation, oxidative stress, and metabolic abnormalities. These acquired pathogenic mechanisms proceed against the background of a hereditary vulnerability, which links mainly to the serotonergic system of the brain and the mechanisms of neurotrophic support. Together, they lead to violations of emotional and cognitive processing. Some of them could be recommended to support the PSD diagnosis, while others need to be clarified before routine clinical use.

Concerning neuroimaging data, we can conclude that ischemic stroke lesion localization, its size, and additional burdening anatomical factors are pathogenically related to PSD. Lesions affecting the frontal-subcortical circles of mood regulation (prefrontal cortex, basal nuclei, and thalamus) predominantly in the left hemisphere can be considered as imaging markers for PSD and also as predictors of PSD development for at least 1 year after the stroke onset. Total anterior cerebral ischemia leads to a higher PSD occurrence; lacunar infarcts less often cause depression symptoms. Additional pontine and lobar cerebral microbleeds in acute stroke patients, as well as severe microvascular lesions of the brain, increase the likelihood of PSD occurrence. Considering that the maximal recovery after stroke is reached within the first year from its onset, it may be recommended to take into account the indicated localizations of stroke lesions in an acute period for preventive and therapeutic strategies of depression.

Resuming the studies on molecular markers, we can distinguish the following candidates, with the help of which PSD patients can be differentiated from non-depressed stroke patients. A significant decrease in serum BDNF concentrations at the early stage of stroke predisposes to the development of PSD. Increased early markers of inflammation (Hs-CRP, ferritin, neopterin, and glutamate), as well as serum pro-inflammatory cytokines (TNF-α, IL-1β, IL-6, IL-18, IFN-γ) and pro-inflammatory/anti-inflammatory ratios (TNF-α/IL-10, IL-1β/IL-10, IL-6/IL-10, IL-18/IL-10, IFN-γ/IL-10) are associated with PSD development. PSD is also characterized by lowered complement expression and decreased serum vitamin D levels. Hypercortisolemia and the blunted cortisol awakening response are the most prominent features of HPA axis dysfunction in PSD. In our view, studies of treatment effects directed on the above mentioned immunological and neuroendocrine mechanisms can validate these markers to be incorporated in routine clinical practice.

Genetic markers, namely S/S 5-HTTLPR, STin2 9/12 and 12/12 genotypes of the serotonin transporter gene SLC6A4, 5-HTR2a 1438 A/A and BDNF met/met genotypes, can reflect the genetic basis for the hereditary predisposition of PSD. To epigenetic markers of PSD, higher SLC6A4 promoter and BDNF promoter methylation status can be assigned.

Neurophysiological markers of PSD, reflecting a violation of perception and cognitive processing, are the elongation of the latency of N200, P300, and N400, as well as a decrease in the P300 and N400 amplitude of the ERPs. The validity of this biological marker should be additionally checked after antidepressant treatment.

In our opinion, taking into account previous remarks, the selected panel of biological markers may be useful for paraclinical underpinning of PSD diagnosis, clarifying various aspects of its multifactorial pathogenesis, optimizing therapeutic interventions, and assessing treatment effectiveness.

The validity of mentioned bellow markers for clinical practice has to be confirmed in further research. Among them are lower 5-hydroxyindoleacetic acid levels in cerebrospinal fluid, increased IL-17 serum levels, elevated serum concentrations of malondialdehyde and oxidative damage of proteins, high serum levels of glucose, uric acid, bilirubin, ApoE, and leptin together with changes in urine concentrations of arabinitol, azelaic acid, formate, glyceric acid, lactate, phenylalanine, pseudouridine, tyrosine, α-hydroxybutyrate, and 5-hydroxyhexanoic acid.

## Author contributions

OL formulated the main concept, searched the literature, and wrote the manuscript. AT provided critical review and revision of the article. Both authors prepared the final version of the manuscript.

### Conflict of interest statement

The authors declare that the research was conducted in the absence of any commercial or financial relationships that could be construed as a potential conflict of interest.
